# Health-Risk Behaviors among Chinese Adults during the COVID-19 Pandemic

**DOI:** 10.3390/ijerph20032157

**Published:** 2023-01-25

**Authors:** William Ho Cheung Li, Laurie Long Kwan Ho, Ankie Tan Cheung, Wei Xia, Peige Song, Joyce Oi Kwan Chung

**Affiliations:** 1 The Nethersole School of Nursing, The Chinese University of Hong Kong, Hong Kong, China; 2 School of Nursing, Sun Yat-Sen University, Guangzhou 510275, China; 3 School of Public Health, Zhejiang University School of Medicine, Hangzhou 310027, China; 4 Centre for Global Health Research, Usher Institute of Population Health Sciences and Informatics, The University of Edinburgh, Edinburgh EH8 9YL, UK; 5 School of Nursing, The Hong Kong Polytechnic University, Hong Kong, China

**Keywords:** COVID-19 pandemic, drinking alcohol, health-related lifestyles, health-risk behaviors, non-communicable diseases, smoking, physical inactivity, unhealthy diet

## Abstract

This study analyzed archived data from a previous large-scale survey study on multiple health-risk behaviors among Chinese adults in Hong Kong between 21 June and 31 August 2021. In addition, this study examined participants’ perceptions of the risks associated with their behaviors, their attitudes toward adopting healthy behaviors, and the impact of COVID-19 on their health-risk behaviors. A total of 4605 participants who had at least one health-risk behavior were included in the analysis. The results showed that about half of the participants were unaware that non-communicable diseases (NCDs) can be caused by health-risk behaviors such as tobacco use, harmful use of alcohol, physical inactivity, and an unhealthy diet. More than half of the participants did not have regular body checkups or monitor their physical health at home. Many participants paid more attention to their health due to the COVID-19 pandemic, but few made changes to their unhealthy habits or adopted a healthier lifestyle. Of the 704 smokers, 60.9% did not realize that smoking increases the risk of transmitting COVID-19 to others. Only 32.2% and 11.9% smokers had the intention to quit smoking and reduced their cigarette consumption, respectively. Additionally, 13.6% reported that their daily cigarette consumption had increased, and 78.8% changed their smoking behavior during the pandemic. Healthcare professionals must educate the public about the association between health-risk behaviors and NCDs and between COVID-19 and NCDs. The government should formulate a long-term plan to strengthen the primary healthcare system and address the challenges posed by the rising prevalence of NCDs.

## 1. Introduction

Non-communicable diseases (NCDs, also referred to as chronic diseases), such as cancer, diabetes, cardiovascular diseases (e.g., heart attacks and strokes), and chronic respiratory diseases (such as chronic obstructive pulmonary disease) kill 41 million people each year, equivalent to 71% of all deaths globally [1]. The high mortality and morbidity rates associated with NCDs greatly impact the health of the affected individuals and place a substantial burden on patients’ families, the healthcare system, the economy, and society [2].

Most NCDs are caused by four health-risk behaviors, namely, tobacco use, harmful use of alcohol, physical inactivity, and an unhealthy diet, and lead to four key metabolic or physiological changes: increased blood pressure, excess weight or obesity, elevated blood glucose, and elevated cholesterol [3]. The World Health Organization (WHO) states that 80% of heart disease, stroke, and diabetes cases can be prevented through lifestyle changes such as quitting smoking, avoiding excessive drinking, and/or adopting healthy behaviors, e.g., eating a balanced diet and engaging in regular physical activity [3]. However, many people lack the motivation or find it difficult to change their health-risk behaviors and adopt healthy lifestyles, especially if they do not have the advice or support of a healthcare professional [4]. Therefore, an innovative approach is needed to motivate more people to change their health-risk behaviors and lead healthier lives. First, however, understanding people’s perceptions of the risks associated with their behaviors and changing their attitudes toward adopting healthy behaviors are important prerequisites for designing innovative and effective interventions to help them lead healthier lives. It is particularly crucial to understand people’s health-risk behaviors and health-related lifestyles during the coronavirus disease 2019 (COVID-19) pandemic, as this has posed considerable health threats and challenges to Hong Kong and the global community.

According to the WHO, tobacco use is a major risk factor for contracting COVID-19, as smoking harms the lungs, and the virus can spread from the fingers to the mouth during smoking [5,6]. A cross-sectional telephone survey (*N* = 201) was conducted through the Youth Quitline Service in 2020 to examine the impact of the COVID-19 pandemic on smoking cessation, smoking behavior, and smoking risk perception among Hong Kong adolescents [7]. The results showed that 68% (*N* = 137) of respondents did not know that smoking increases the risk of transmitting COVID-19. About 43% (*N* = 86) of respondents said they intended to quit smoking because of the pandemic. Moreover, approximately 58% (*N* = 117) of respondents reported that they had reduced their tobacco use. In addition, the Youth Quitline Service in Hong Kong reported a 43% increase in incoming calls from those seeking help to quit smoking during the pandemic and a 26.2% to 38% increase in biochemically validated quit rates [7]. The findings of this study highlighted the positive impact of COVID-19 on motivating young Chinese smokers to quit, thereby improving their health and reducing the risk of spreading COVID-19. However, it is uncertain whether the effects of the COVID-19 pandemic are having the same impact on Chinese adult smokers. Furthermore, it is uncertain whether the pandemic is a good opportunity to motivate people to change health-risk behaviors other than smoking or to adopt healthy lifestyles.

The residents of Hong Kong, like those of many other countries, face an increasing risk of developing NCDs [8]. We conducted a large-sample survey (*N* = 5737) of Hong Kong Chinese adults in 2021 to investigate the prevalence and clustering patterns of multiple health-risk behaviors [8]. The results showed that 80.3% (*N* = 4605) of the respondents had at least one health-risk behavior. Furthermore, the survey provided some evidence that health-risk behaviors co-occur in clusters. Multiple regression analysis indicated that smoking, alcohol consumption, and the number of health-risk behaviors were factors associated with NCDs [8]. This paper further reports on the respondents’ perceptions of the risks related to their behaviors and their attitudes toward adopting healthy behaviors. This study aims to examine participants’ perceptions of the risks associated with their behaviors and their attitudes toward adopting healthy behaviors. Additionally, this study describes the impact of the COVID-19 pandemic on the health-risk behaviors and health-related lifestyles of Hong Kong residents. 

## 2. Materials and Methods

### 2.1. Design and Sample

We analyzed archived data from our previously published, cross-sectional, descriptive study on multiple health-risk behaviors among Chinese adults [8]. The study [8] was conducted in all 18 districts of Hong Kong from 21 June to 31 August 2021. The design, sampling, and recruitment methods were reported in detail in our previously published paper [8]. Ethical approval was obtained from the Institutional Review Board of the University of Hong Kong/Hospital Authority, Hong Kong West Cluster (reference number: UW21-440). Informed written consent was obtained from each eligible participant after the purpose of the study was explained. The participants were told that they had the right to withdraw from the study at any time and were assured of the confidentiality of their data. This study adhered to the *Declaration of Helsinki: Ethical Principles for Medical Research Involving Human Subjects* (https://www.wma.net/policies-post/wma-declaration-of-helsinki-ethical-principles-for-medical-research-involving-human-subjects/, accessed on 20 December 2022). 

### 2.2. Measures

A behavioral risk factor questionnaire was used to collect the eligible participants’ demographic and clinical data and information on their health-risk behaviors. This questionnaire was adapted from a Hong Kong Department of Health document (www.chp.gov.hk/files/pdf/brfs_2015apr_en.pdf, accessed on 5 January 2021) and modified to investigate multiple health-risk behaviors among 5737 Chinese adults in Hong Kong in 2021 [8]. In addition, a set of structured questions was added to the end of the questionnaire to assess participants’ perceptions of the risks associated with their behaviors, their attitudes toward adopting healthy behaviors, and the impact of COVID-19 on their health-risk behaviors. The criteria for determining whether the person engaged in each health-risk behavior were clearly mentioned in our previous paper [8].

### 2.3. Data Analysis

SPSS for Windows (SPSS version 26.0; IBM Corp., Armonk, NY, USA) was used for data analysis. Descriptive statistics were used to calculate the mean, standard deviation, and frequency of the demographic and health-risk behavior data. Chi-square tests for independence were used to compare any differences in health-risk behaviors between participants with and without NCDs.

## 3. Results

In the survey reported in our previous paper [8], 5737 people participated, 4605 of whom had at least one health-risk behavior. We analyzed the findings of the study for those participants who engaged in health-risk behaviors. The demographic characteristics of the participants are shown in Table 1. More men (53.9%) than women (46.1%) engaged in health-risk behaviors. More than half of the participants were between the ages of 30 and 50 (62.4%) and married (67.6%). Most participants (75.2%) had completed their education to upper secondary school level or above. Referring to the body mass index classification of Chinese adults that has been adopted in Hong Kong, 16.3% and 17.8% of participants were classified as overweight and obese, respectively. Of the 4605 participants, 15.3%, 15.9%, 51.1%, and 94% were smokers, drinkers, consumers of unhealthy diets, and physically inactive, respectively. 

Table 2 compares differences in health-risk behaviors between participants with and without NCDs. There were no statistically significant differences regarding alcohol consumption, unhealthy diets, or physical inactivity between participants with and without NCDs. However, there was a statistically significant difference in smoking status between the two groups, with the NCD group containing more smokers. 

Table 3 shows the responses of participants to the closed-ended items in the questionnaire used to assess the risk perceptions of health-risk behaviors, adopting healthy behaviors, and the impact of COVID-19 on health-risk behaviors. 

### 3.1. Risk Perceptions of Health-Risk Behaviors

The results showed that almost half of the participants (48.4%) were unaware that NCDs can be caused by health-risk behaviors such as tobacco use, harmful use of alcohol, physical inactivity, and an unhealthy diet. Furthermore, more than half of the participants (61.9%) did not believe that quitting health-risk behaviors would help prevent NCDs.

### 3.2. Adopting Healthy Behaviors

The findings indicated that many participants wanted to prevent NCDs by adopting a healthy lifestyle. However, less than half of the participants (42.1%) had had a health checkup in the past three years, and only 36.9% regularly monitored their physical health at home. 

### 3.3. Impact of COVID-19 on Health-Risk Behaviors

The results showed that many participants (68.2%), indeed, paid more attention to their health due to the COVID-19 pandemic. However, only 28.3% participants had made changes to their unhealthy habits or adopted a healthier lifestyle. For those who smoked, more than half (60.9%) believed that smoking does not increase the risk of spreading COVID-19 to their friends and families. Only 32.2% of smokers claimed that the pandemic had increased their intention to quit smoking, and 11.9% had reduced their cigarette consumption. However, 13.6% of smokers reported that their daily cigarette consumption had increased during the pandemic. In addition, many smokers (78.8%) claimed that COVID-19 and the pandemic prevention measures had affected their smoking behavior. 

## 4. Discussion

Hong Kong is facing a growing threat from NCDs that is intensified by the city’s ageing population [9]. Failure to reduce this threat will ineluctably lead to increased mortality and morbidity. Immediate attention is needed to develop and evaluate effective interventions that promote lifestyle changes to prevent people from developing NCDs [10]. This study addressed some literature gaps by investigating Chinese people’s perceptions of health-risk behaviors and attitudes toward adopting healthy behaviors. These data are necessary prerequisites for designing appropriate and innovative interventions to help people with unhealthy habits lead healthier lives. A large-scale survey was conducted across all 18 districts in Hong Kong during the COVID-19 pandemic, as it is crucial to study how the pandemic is affecting people’s health-risk behaviors.

The results showed that a very high proportion of participants (94%) did not meet the WHO’s adult physical activity recommendations of at least 75 min per week of vigorous-intensity or 150 min of moderate-intensity aerobic physical activity [11]. Although it is well documented that many Hong Kong adults have more sedentary lifestyles than their Western counterparts [12], caution must be taken in interpreting the results. First, data collection took place during the COVID-19 pandemic, when wearing a mask was mandatory in all public areas. Because wearing a face covering can lead to difficulty breathing, many people may have been hesitant to engage in moderate-to-vigorous exercise outdoors under these restrictions. Second, all indoor sports facilities, such as sports centers, badminton halls, squash courts, swimming pools, and gyms, were closed, making it more difficult for people to exercise. Therefore, this study may have overestimated the proportion of physically inactive individuals among Hong Kong Chinese adults. However, as physical inactivity is one of the leading causes of NCDs and premature deaths globally [13], governments and healthcare professionals should make more effort to promote the importance of engaging in regular physical activity, even during the COVID-19 pandemic.

In addition to alcohol consumption, an unhealthy diet, and physical inactivity, smoking was also linked to NCDs, as the results showed there were significantly more smokers with NCDs than without NCDs. One possible reason for this is that smoking plays an important causal role in the development of NCDs [14]. This argument is consistent with our previous findings that, among all health-risk behaviors, smoking is the strongest factor associated with NCDs [8]. Given that 14% of all NCD-related deaths worldwide are attributable to smoking [15], and evidence shows that continued smoking by persons with NCDs reduces the efficacy of treatment and increases the risk of side effects [16,17], it is paramount that healthcare professionals develop innovative and effective smoking cessation interventions to help smokers, especially those with NCDs, to quit smoking.

Despite the evidence showing that NCDs are caused by tobacco use, harmful use of alcohol, physical inactivity, and an unhealthy diet, this study showed that almost half of the participants were unaware of the relationships. Furthermore, many participants were unaware that NCDs can be prevented through lifestyle changes [18]. Some participants mistakenly believed that NCDs are caused by genetics, are unrelated to healthy behaviors and lifestyles, and are, therefore, inevitable. It is vital that the Hong Kong government and healthcare professionals correct these misconceptions and step up efforts to advocate the importance of healthy behaviors and lifestyles in the prevention of NCDs [3,19]. 

Although many participants expressed a desire to live a healthy life and reduce their risk of NCDs, many of them did not act on it. Consistent with the findings of a previous study [20], this study showed that many Chinese adults do not undergo regular physical examinations or take actions, such as measuring blood pressure, weight, and blood sugar, to monitor their own health at home. Although regular body checkups are important preventive measures to manage NCDs [21], many Chinese adults mistakenly believe that physical examinations are not necessary if they are not feeling unwell [19]. It is essential for healthcare professionals to correct the public’s misconceptions and educate them on the benefits of regular checkups for the management of NCDs. Most importantly, it is imperative that the government develops a community-based primary healthcare system that closely monitors citizens for the prevention and management of NCDs. The Hong Kong government issued the “Primary Healthcare Blueprint” on 19 December 2022, outlining their plan to strengthening the primary healthcare system and address the challenges brought about by the rising prevalence of NCDs (https://www.info.gov.hk/gia/general/202212/19/P2022121900561.htm, accessed on 21 December 2022).

Because of the COVID-19 pandemic, many people’s concerns for their own health have increased. Nevertheless, the majority of participants (71.7%) said that they have not changed their unhealthy habits or adopted a healthier lifestyle. A possible reason is that most people did not understand the relationship between COVID-19 and NCDs, and they paid more attention to infection control to prevent contracting the disease. Evidence suggests that people with NCDs are particularly vulnerable to COVID-19 and are at a higher risk of developing severe symptoms [21,22,23,24]. In particular, smokers infected with COVID-19, especially those with NCDs, are at a higher risk of severe complications and death than non-smokers [5,22,25].

Consistent with our previous study examining the impact of the COVID-19 pandemic on smoking risk perceptions among Hong Kong adolescents [7], this study found that many Chinese adults were unaware that tobacco use increases the risk of transmitting COVID-19 to others. In addition, a recent study found a causal link between smoking and the risk of contracting mild-to-severe COVID-19 [26]. This issue cannot be overlooked because the health risks of tobacco come not only from direct smoking but also from exposure to second-hand smoke [27], especially during the COVID-19 pandemic, as the smoke can transmit the virus to families and friends [7,28]. 

Unlike the findings of a previous study [7], in which 43% of young smokers were motivated to stop smoking, only 32.2% of adult smokers in this study reported that the COVID-19 pandemic motivated their decision to quit. Only 11.9% of adult smokers in this study reduced their daily cigarette consumption during the COVID-19 pandemic, compared to 58% in the previous study [7]. In addition, 13.6% of adult smokers in this study had increased their daily cigarette consumption. There are some possible reasons for these findings. First, similarly to China, Hong Kong adopted a zero-COVID policy and strenuous epidemic prevention measures to contain the transmission of COVID-19 and keep the number of cases as close to zero as possible. To prevent clusters of infection in the community, the Hong Kong government ordered the closure of movie theaters, karaoke bars, bars, and pubs in March 2020. Gatherings of more than four people were prohibited, and other social distancing rules were enforced to prevent people from congregating in public places. In addition, people were required to wear masks outdoors and in public places. All these stringent precautions had a particular impact on young smokers, who had to stay home because of school closures during the data collection period and whose parents were largely unaware of their smoking habits. In our previous study [7], some young smokers expressed their desire to quit smoking because of the need to wear a mask, which made it harder to smoke in public areas; the inability to smoke at home; and a lack of peer pressure to smoke. Second, unlike the majority of young smokers, who tend to be occasional smokers with mild nicotine dependency [29], many adult smokers are chronic smokers with moderate-to-high nicotine dependency [30]. In fact, cigarette smoking is so addictive that many chronic smokers either have no intention of quitting or have difficulty quitting [28]. Third, unlike young smokers, adult smokers had greater flexibility that allowed them to find areas to smoke when rigorous infection prevention measures were in place, such as in the hallways or toilets in the workplace and even at home. This explains why many adult smokers reported that the pandemic and anti-epidemic measures affected their smoking behaviors. In addition, as there was more time spent at home and less time spent outdoors during the COVID-19 pandemic, some adult smokers reported smoking more than usual because of boredom. Over the past few decades, the Hong Kong government and healthcare professionals have made enormous efforts to advocate for raising the tobacco tax, introduce legislation, law enforcement, and organize anti-smoking campaigns to reduce the smoking prevalence [31]. However, given that the findings of this study show that many smokers lack a good understanding of the relationship between smoking and NCDs and between smoking and COVID-19, it is also imperative that the government develops smoking cessation interventions with a focus on enhancing smokers’ understanding of these relationships. This will not only help to improve the physical well-being of smokers with NCDs but also protect the public, especially vulnerable groups such as women and children, from exposure to second-hand smoke. This will ultimately save more lives, protect the environment, and support sustainable development.

As reported in our previous paper [8], the study had several limitations, including potential response bias, a lack of objective measures for NCDs, and an inability to verify the causality of variables because of its cross-sectional nature. In addition, this study was limited by its use of only one set of structured questions to gain insights into how participants perceived the risks associated with their behaviors, their attitudes toward adopting healthy behaviors, and the impact of COVID-19 on their health. An in-depth qualitative interview with open-ended questions should be conducted in future studies. 

## 5. Conclusions

This study examined Hong Kong Chinese adults’ perceptions of the risks related to their behaviors and their adoption of healthy behaviors, especially during COVID-19, and of their risk behaviors and health-related lifestyles, which are underrepresented in the existing literature. The results showed that many Hong Kong Chinese adults lack a good understanding of the relationships between health-risk behaviors and NCDs and the relationship between COVID-19 and NCDs. It is essential that healthcare professionals put more effort into promoting a healthy lifestyle and informing the public of its importance in preventing and managing NCDs. In addition, it is vital to educate the public about the association between COVID-19 and NCDs. Most importantly, the government should formulate a long-term plan to strengthen the primary healthcare system and address the challenges brought about by the rising prevalence of NCDs.

## Figures and Tables

**Table 1:** Demographic characteristics of the participants with at least one health-risk behavior (*N* = 4605) ^a^.

Characteristic	Weighted Sample % (No. of Participants)
Age (years)	
30 to 40	36.8 (1695)
41 to 50	25.6 (1179)
51 to 60	16.7 (769)
61 to 70	12.7 (585)
71 to 80	6.0 (276)
81 to 90	2.2 (101)
Sex	
Male	53.9 (2482)
Female	46.1 (2123)
Marital status	
Single	27.6 (1262)
Married or cohabiting	67.6 (3091)
Divorced, separated, or widowed	4.8 (220)
Educational attainment	
Primary school or below	10.7 (489)
Lower secondary school	14.1 (645)
Upper secondary school	44.9 (2053)
Tertiary education	30.3 (1386)
Household income	
HKD <20,000	12.5 (572)
HKD 20,000–39,999	30.6 (1399)
HKD 40,000–59,999	26.0 (1189)
HKD 60,000 or above	30.0 (1372)
Employment status	
Unemployed or retired	29.6 (1354)
Employed	70.4 (3219)
Body mass index classification	
Underweight (<18.5)	11.4 (525)
Normal (18.5 to 23.0)	54.5 (2509)
Overweight (23.0 to 25.0)	16.3 (751)
Obese (>25)	17.8 (820)
Non-communicable diseases	
No	72.7% (3347)
Diabetes	10.8% (497)
Chronic respiratory diseases	3.2% (148)
Cancer	0.9% (42)
Cardiovascular diseases	12.4% (571)
Health-risk behaviors	
Tobacco use	
Smokers	15.3% (704)
Non-smokers	84.7% (3901)
Alcohol use	
Drinkers	15.9% (734)
Non-drinkers	84.1% (3882)
Diet intake	
Unhealthy diet intake	51.1 (2353)
Healthy diet intake	48.9 (2252)
Physical activity	
No regular physical activity	94.0 (4330)
Have regular physical activity	6.0 (275)

^a^ Sample sizes varied because of missing data on some variables.

**Table 2:** Differences in health-risk behaviors between participants with and without non-communicable diseases.

	Non-Communicable Disease Yes (*N* = 1258)	Non-Communicable Disease No (*N* = 3347)	χ^2^	*p*
Smoking	271 (21.5)	433 (12.9)	51.62	<0.001
Alcohol consumption	204 (16.2)	530 (15.8)	0.07	0.79
Unhealthy diet	663 (50.3)	1690 (50.5)	1.70	0.19
Physical inactivity	1187 (94.4)	3143 (93.9)	0.26	0.61

**Table 3:** Participants’ risk perceptions of health-risk behaviors, adopting healthy behaviors, and impact of COVID-19 on health-risk behaviors.

Questions	Responses, *N* (%)	
	Yes	No
	(*N* = 4605)	
Risk perceptions of health-risk behaviors		
1. Do you know that non-communicable diseases can be caused by health-risk behaviours, such as tobacco use, harmful use of alcohol, physical inactivity, and unhealthy diet?	2376 (51.6)	2229 (48.4)
2. Do you think quitting health-risk behaviors can help prevent non-communicable diseases?	1753(38.1)	2852 (61.9)
Adopting healthy behaviors		
1. Are you willing to build a healthy life to prevent non-communicable diseases?	3619 (78.6)	986 (21.4)
2. In the past three years, have you had a health check?	1937 (42.1)	2668 (57.9)
3. Do you often monitor your physical condition at home, such as measuring blood pressure, blood sugar, weight?	1700 (36.9)	2905 (63.1)
Impact of COVID-19 on health-risk behaviors		
1. Are you paying more attention to your health due to the COVID-19 pandemic?	3139 (68.2)	1466 (31.8)
2. As a result of the COVID-19 pandemic, have you changed unhealthy habits or adopted a healthier lifestyle?	1303 (28.3)	3302 (71.7)
	(*N* = 704)	
3. * Do you think tobacco use will increase the risk of spreading COVID-19 to your family or friends?	275 (39.1)	429 (60.9)
4. * Has the pandemic increased your intention to quit smoking?	227 (32.2)	477 (67.8)
5. * Has your daily cigarette consumption decreased during the pandemic?	84 (11.9)	620 (88.1)
6. * Has your daily cigarette consumption increased during the pandemic?	96 (13.6)	608 (86.4)
7. * Have the pandemic and the anti-epidemic measures affected your smoking behavior?	555 (78.8)	149 (21.2)

* Questions for smokers only.

## Data Availability

The data presented in this study are available on request from the corresponding author. The data are not publicly available due to privacy and ethical concerns.
